# Short-wave infrared (SWIR) spectroscopy and imaging of biological tissues: a decade of advancements (2016-2025)

**DOI:** 10.1117/1.JBO.31.1.010901

**Published:** 2025-12-15

**Authors:** Robert H. Wilson, Gordon T. Kennedy, Christopher A. Campbell, Thinh Phan, Alex Hao Lin, Benjamin Levi, Anthony J. Durkin

**Affiliations:** a University of Dayton, Department of Physics, Dayton, Ohio, United States; b University of California, Irvine, Beckman Laser Institute, Irvine, California, United States; c University of Texas Southwestern, Department of Surgery, Dallas, Texas, United States

**Keywords:** short-wave infrared, short-wave infrared, spectral imaging, tissue optics, tissue spectroscopy

## Abstract

**Significance:**

Short-wave infrared (SWIR) light has recently gained popularity in tissue spectroscopy and imaging applications for a wide range of biomedical applications, primarily due to advancements in hardware (e.g., cameras).

**Aim:**

We aim to provide a detailed review of SWIR-based biomedical optics studies from the past decade, during which there has been a proliferation of SWIR-based tissue-optics studies.

**Approach:**

We report literature occurring after the publication of our previous (2015) review of this space, describing next-generation SWIR-based techniques that hold significant promise for enhanced *in vivo* tissue characterization and clinical translation.

**Results:**

Interest from the biophotonics field in SWIR technology is typically attributable to (1) the capability of SWIR light to provide greater sensitivity to chromophores such as water and lipids, with absorption peaks not as prominent in the visible-to-near-infrared (VIS-NIR) spectral region, and (2) the potential for SWIR photons to penetrate through superficial tissue layers due to lower scattering in the SWIR than in the VIS-NIR, as well as substantially reduced attenuation from hemoglobin and melanin.

**Conclusion:**

This review of emerging SWIR biophotonic technologies illustrates the rapid growth in the use of SWIR light for *in vivo* tissue spectroscopy and imaging.

## 1 Introduction

Optical spectroscopy and imaging devices have commonly relied on visible-to-near-infrared (VIS-NIR) light to interrogate the physiological properties of biological tissue.^1^ Light sources are abundant in this wavelength range and include broadband incandescent lamps, light-emitting diodes (LEDs), and lasers. In addition, this wavelength range corresponds to some degree with the bandgap of silicon, making silicon technology relatively inexpensive and commonplace for the detection of VIS-NIR photons.^2^ The VIS-NIR range (∼400 to 1000 nm) provides high sensitivity to oxygenated and deoxygenated hemoglobin,^3^ making it well suited for quantifying the oxygenation state of tissue. Absorption $\left[\mu_{a}(\lambda )\right]$ and reduced scattering $\left[\mu_{s}^{\prime }(\lambda )\right]$ spectra measured in the VIS-NIR have been analyzed by numerous groups over the past 30+ years to extract parameters that can be employed for the identification and monitoring of tissue disease, injury, and recovery.^1^ However, VIS-NIR systems are subject to several notable limitations. First, several physiologically important tissue chromophores, including water^4^ and lipids,^5^^,^^6^ do not have as strong absorption peaks in the VIS-NIR, making it difficult to reliably quantify changes in the concentrations of these chromophores. Second, the tissue reduced scattering coefficient [$\mu_{s}^{\prime }(\lambda )$; Ref. 7] and the absorption coefficient of melanin [$\mu_{a\_\mathrm{mel}}(\lambda )$; Ref. 8] both decrease monotonically with wavelength, and the absorption coefficient of hemoglobin [$\mu_{a\_\mathrm{Hb}}(\lambda )$; Ref. 3] is also substantially greater at visible wavelengths. Therefore, high scattering and absorption cause many VIS-NIR wavelengths to only interrogate very superficially into the tissue, making it difficult to quantify (or image) physiological changes deeper beneath the surface.

Short-wave infrared (SWIR) light, ranging from ∼1000 to 2000 nm, has recently gained popularity in the biophotonics field due to its potential to overcome the limitations of VIS-NIR wavelengths described above.^9^^–^^11^ Water (near 1150, 1450, and 1900 nm) and lipids (near 1040, 1200, 1400, and 1700 nm) have strong absorption features in the SWIR,^4^^–^^6^ allowing the use of SWIR to facilitate a more sensitive and accurate quantification of physiological changes related to perturbations in water and lipid concentrations. Given that $\mu_{a\_\mathrm{mel}}(\lambda )$, $\mu_{a\_\mathrm{Hb}}(\lambda )$, and $\mu_{s}^{\prime }(\lambda )$ are all lower in the SWIR than in the VIS-NIR,^3^^,^^7^^,^^8^ melanin, hemoglobin, and tissue scattering are not expected to substantially attenuate the incident light, limit the penetration depth, or confound the sensitivity of the detected signals to absorption contrast from water and lipids. Therefore, SWIR techniques are believed to be well suited for spectroscopy and imaging of a wide range of tissue types, with enhanced ability to quantify water and lipid content.

In 2015, a decade before this current report, our group published a review of SWIR-based tissue optics studies,^9^ summarizing the work done in this area as SWIR techniques were beginning to gain a footing in the biophotonics community. The 2015 review provided a preliminary illustration of some of the potential advantages afforded using SWIR light to characterize tissue, notably, the increased sensitivity to absorption from water and lipids. However, at the time of that initial review, the set of published studies was much more limited, both in depth and breadth, than the literature that exists currently (as of April 2025). In particular, in the decade since the 2015 review, there have been dramatic advancements in the application of SWIR techniques to *in vivo* tissue characterization and monitoring, and there has also been a rapid increase in the development of tissue measurement techniques that merge SWIR light with other emerging technologies, such as hyperspectral imaging and fluorescent quantum dots. In addition, there had previously been limitations on the ability of researchers to work with Indium Gallium Arsenide (InGaAs) SWIR sensors due to regulations governing the export and import of defense-related articles and services. In the intervening time period, many of those regulations have been relaxed. Therefore, now is a suitable time to report a review of the literature on SWIR-based tissue optics techniques that have been published since the time of our first (2015) review paper. This updated review of emerging SWIR biophotonic technologies over the past decade illustrates the rapid growth in popularity of using SWIR light to facilitate *in vivo* tissue spectroscopy and imaging techniques with enhanced contrast and penetration depth, for a wide range of biomedical applications.

## 2 *In Vivo* SWIR Spectroscopy and Imaging: Quantifying Optical Properties of Skin

Several recent studies^12^^–^^18^ have used SWIR wavelengths to quantify water content, lipid content, and tissue scattering of skin *in vivo* in human volunteers. These studies have also included sensitivity analyses to quantify the degree to which water and lipid perturbations can be quantified via the measured backscattered reflectance, $\mu_{a}$, and $\mu_{s}^{\prime }$ spectra. Selected studies are described in further detail below.

**Fig. 1:**
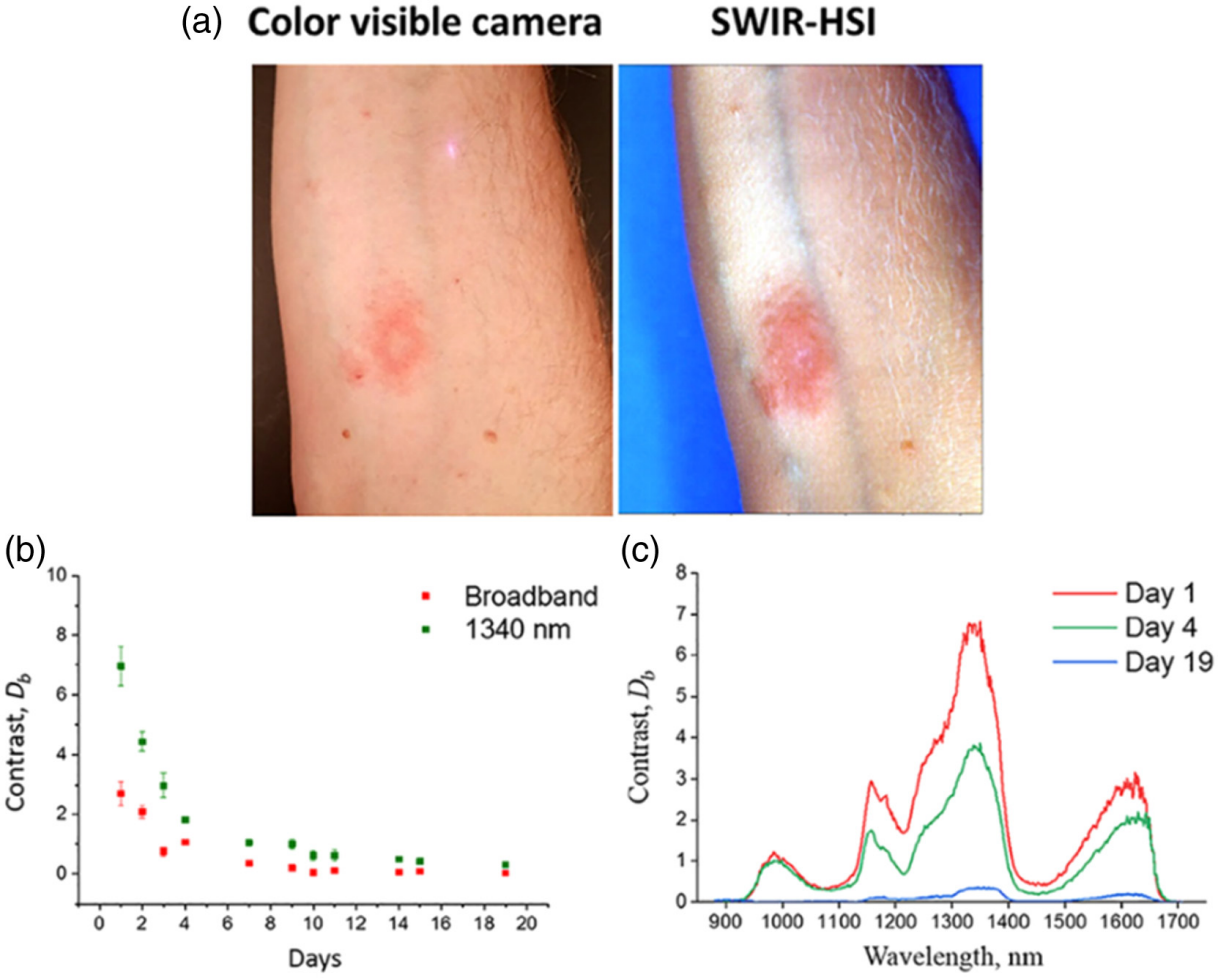
Hyperspectral SWIR imaging provides enhanced contrast, relative to conventional color imaging, for identifying and monitoring contact dermatitis *in vivo*. (a) RGB image from a conventional color camera, compared with RGB mimic constructed from SWIR hyperspectral imaging data, shows enhanced penetration depth and contrast between the inflamed region and surroundings when SWIR data are used. (b) Data from 1340 nm provide increased contrast between the inflamed and noninflamed regions, relative to using broadband data from the SWIR device. (c) Contrast from hyperspectral SWIR imager as a function of wavelength peaks near 1340 nm, with secondary peaks around 1000, 1200, and 1600 nm, enabling monitoring of healing over time. Figure panels were reproduced from Ref. 13 with permission; © 2020 Wiley‐VCH Verlag GmbH & Co.

Ezerskaia et al.^12^ used backscattered light from laser diodes at 1720, 1750, and 1770 nm, detected via a photodiode, to assess the ratio of absorption from lipid to absorption from water in the skin (forehead) of a human volunteer undergoing a tape-stripping procedure. Comparisons were performed with confocal Raman spectroscopy to confirm the accuracy of the measured water and lipid absorption ratios. The study also compared the aforementioned SWIR-light-based approach with commercial devices (Courage & Khazaka Sebumeter^19^ and Corneometer^20^) and found that the SWIR light was more sensitive to lipids than the Sebumeter and more sensitive to water than the Corneometer. Du et al.^13^ employed a hyperspectral imager to obtain backscattered reflectance datacubes (i.e., 2D images at each wavelength), spanning 510 wavelengths from 881 to 1710 nm, from the arm of a person experiencing contact dermatitis following exposure to poison ivy (Fig. 1). The imaging device consisted of an InGaAs CCD camera, a spectrograph, two broadband light sources (halogen lamps), and a translational stage for “push-broom” scanning. Instead of using a reflectance standard to convert the measured data into calibrated reflectance, the contrast at each wavelength between inflamed and noninflamed regions of the skin was calculated, and the wavelengths that provided the greatest contrast were selected for further analysis. Images at three optimal wavelengths (1070, 1340, 1605 nm) were combined to create a SWIR analog of an RGB (digital color) image of the arm on multiple days over a 3-week period following initial development of contact dermatitis. Using the “SWIR-RGB” images, as opposed to images from an individual SWIR wavelength band, provided enhanced sensitivity to the contrast between the lesion and the surrounding tissue throughout the 3-week monitoring period. The “SWIR-RGB” images were also better than conventional RGB images at resolving the lesion throughout the monitoring period, including days when the lesion was difficult to distinguish from the surrounding tissue using conventional RGB imaging.

**Fig. 2:**
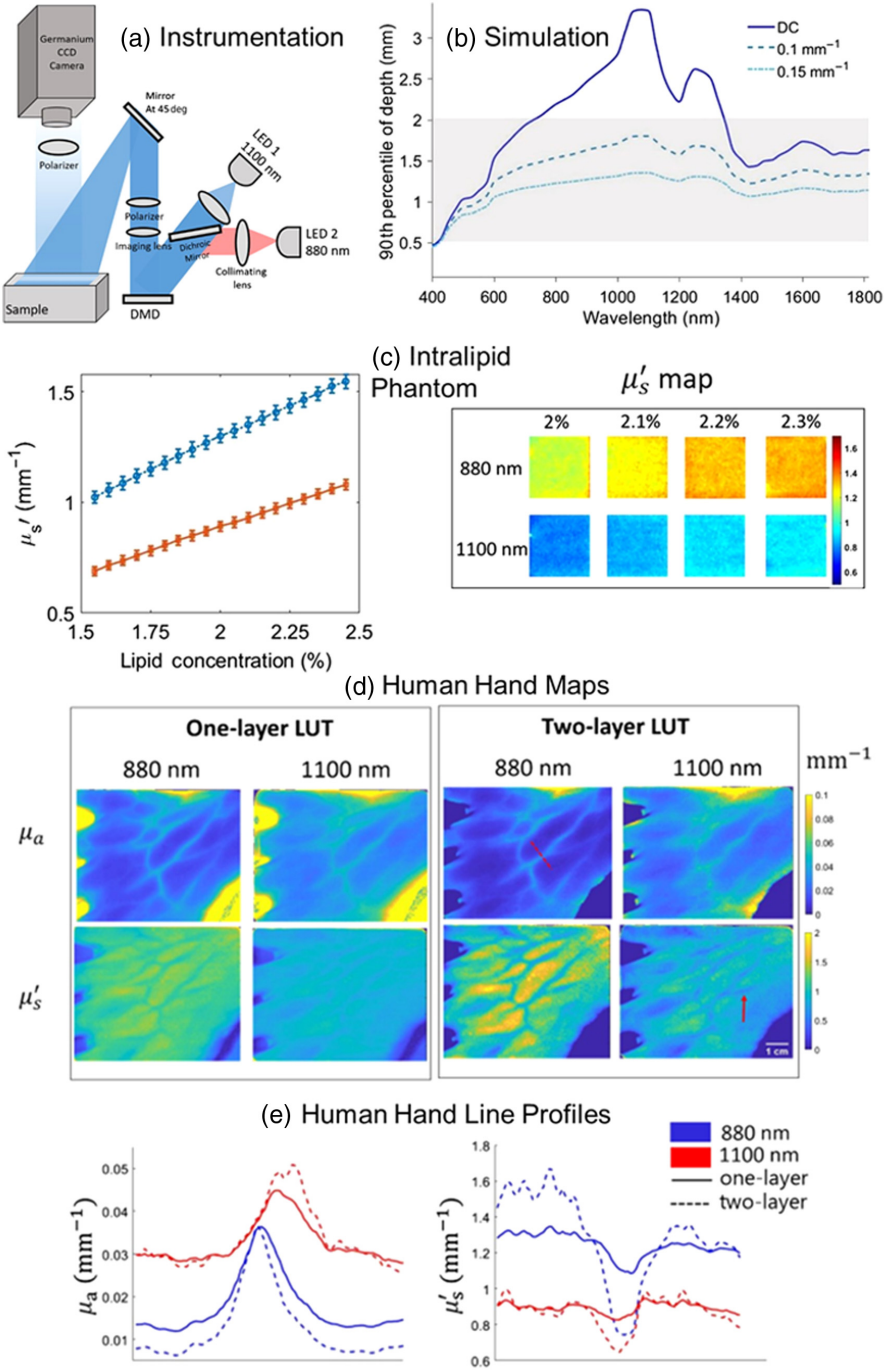
NIR-SWIR SFDI device provides enhanced penetration depth and high sensitivity to small changes in tissue water and lipid fractions. (a) Instrumentation for NIR-SWIR SFDI, using two LEDs (880 and 1100 nm), a light projection system, and a camera with a germanium CCD. (b) Simulation results indicate that penetration depth is enhanced at the beginning of the SWIR regime (∼1000 to 1200 nm). (c) Measured reduced scattering coefficients ($\mu_{s}^{\prime }$) from tissue-simulating phantoms consisting of mixtures of Intralipid and water, showing high sensitivity of $\mu_{s}^{\prime }$ [at both 880 nm (blue data points) and 1100 nm (red data points)] to small changes in the water-to-lipid ratio. (d), (e) Maps and line profiles of absorption and reduced scattering coefficients $\mu_{a}$ and $\mu_{s}^{\prime }$ for the *in vivo* human hand, comparing the use of one-layer and two-layer look-up tables for data analysis. Interestingly, the $\mu_{s}^{\prime }$ and $\mu_{a}$ maps both provided substantial contrast between visible vasculature and surrounding regions. Figure panels were reproduced from Ref. 14 under a CC-BY license.

Pilvar et al.^14^ (Fig. 2) used a spatial frequency domain imaging (SFDI) system with one SWIR wavelength (1100 nm) and one NIR wavelength (880 nm) to produce $\mu_{a}$ and $\mu_{s}^{\prime }$ maps of the hand of a human volunteer. The device contained LEDs at the two wavelengths (880 and 1100 nm), coupled to a digital micromirror device (DMD) to project spatially modulated patterns onto the tissue, with a germanium camera sensitive to wavelengths from ∼300 to 1600 nm.

Figures 2(d) and 2(e) show that using a look-up table (LUT) comprised of two-layered SWIR tissue models, instead of a LUT comprised of homogeneous SWIR tissue models, substantially increased the contrast from large blood vessels in the $\mu_{a}$ and $\mu_{s}^{\prime }$ maps. Surprisingly, they also noticed that certain small features of the vasculature in the hand could be seen more clearly in the $\mu_{s}^{\prime }$ map than in the $\mu_{a}$ map. Furthermore, they demonstrated that the $\mu_{s}^{\prime }$ maps at both wavelengths were sensitive to small (<5%) changes in lipid concentration in phantoms consisting of Intralipid and water. As a component of their study, they used an algorithm based on Monte Carlo simulations to calculate the estimated depth that photons at a range of SWIR wavelengths would be expected to penetrate the tissue. This approach indicated that the un-modulated (DC, $0\;{\mathrm{mm}}^{-1}$) light from wavelengths ∼900 to 1300 nm should penetrate the deepest, reaching subdermal layers of tissue, whereas structured light patterns in that same wavelength range with spatial frequencies of 0.1 and $0.15\;{\mathrm{mm}}^{-1}$ should be confined to the epidermis and dermis. This result suggests that SFDI with SWIR wavelengths may facilitate the separation of dermal and subdermal components of the detected backscattered reflectance and corresponding $\mu_{a}$ and $\mu_{s}^{\prime }$ values.

Spink et al.^15^ designed a wearable optical sensor consisting of 980, 1200, and 1300 nm LEDs, a photodiode for detecting backscattered light, an HDMI cable attaching the sensor to a microcontroller, and a strap for attaching the device to the arm of a patient. The device was shown to accurately quantify the water and lipid fractions within a tissue-simulating phantom. Thus, the study suggested that it would be feasible to develop a low-cost wearable SWIR-based device for monitoring water and lipid content in active patients.

In a 2025 report, Livecchi et al.^16^ developed a system for SFDI that used SWIR-range LEDs (970, 1050, and 1200 nm) in conjunction with a light projection system and a SWIR-sensitive InGaAs camera. The tissue-simulating phantom used for calibrating the measurements was a mixture of Intralipid and water (10% Intralipid), with the “ground truth” values of the phantom’s reduced scattering coefficient $\mu_{s}^{\prime }$ and absorption coefficient $\mu_{a}$ at the three aforementioned wavelengths taken from previous literature.^21^^–^^23^ The resulting calibrated reflectance data were employed to extract $\mu_{a}$ and $\mu_{s}^{\prime }$ at each wavelength, as well as the water fraction of the sample. The performance of the system was assessed by measuring *ex vivo* porcine skin over the course of a desiccation process (to modify the water content), as well as the hand *in vivo*, pre- and post-exercise, in a human volunteer (to assess the effect of sweating on tissue hydration). Changes in the water content of tissue were found to impact not only the tissue water fraction but also the values of $\mu_{s}^{\prime }$ at the three wavelengths, indicating that $\mu_{s}^{\prime }$ may be an additional important parameter for quantifying the hydration of tissue. This finding was similar to that recently reported by our group for an *in vivo* porcine burn model in which hydration-related changes in tissue physiology were interrogated using VIS-NIR spatial frequency domain imaging.^24^

**Fig. 3:**
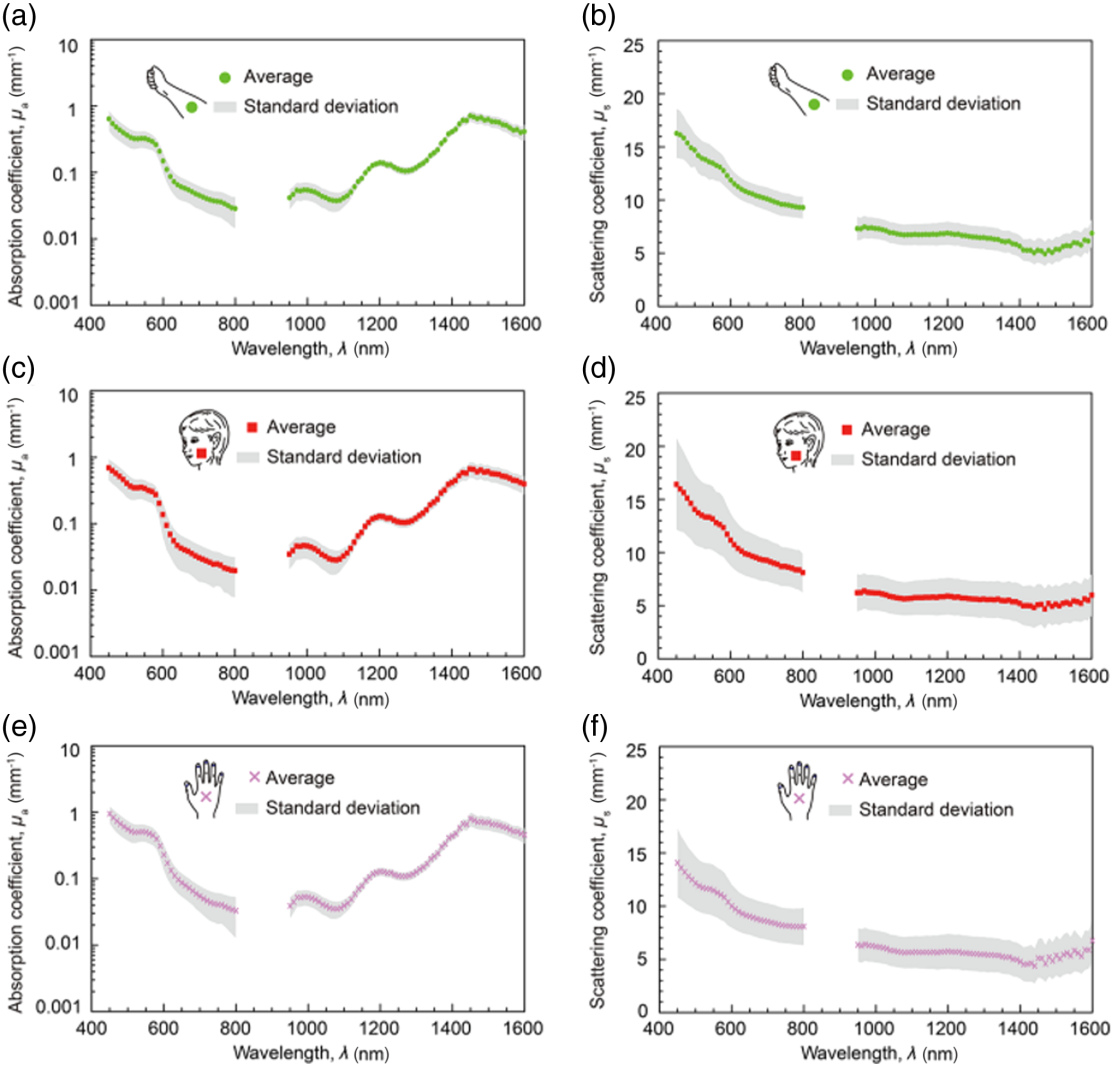
Human skin tissues measured in the SWIR *in vivo* at multiple anatomical locations exhibit absorption peaks characteristic of water and lipids (a), (c), (e) and a continuation of the inverse-power-law scattering behavior seen in the VIS-NIR (b), (d), (f). Measured absorption spectra from the forearm, cheek, and palm, shown as averages and standard deviations over 198 subjects, had peaks near 970 and 1450 nm (attributable to water) as well as a peak near 1200 nm (attributable to lipids). By contrast, the main observable VIS-NIR absorption features (∼450 to 600 nm) were due to hemoglobin. Measured scattering spectra from these same anatomical locations, averaged over the same set of subjects, exhibited a systematic decrease with wavelength that was flatter in the SWIR than in the VIS-NIR but appeared to form a continuation of the inverse-power-law trend seen for VIS-NIR tissue scattering. Figure panels were reproduced and modified from Ref. 17 under a CC-BY license.

Kono and Yamada^17^ used a broadband light source and a mask composed of a periodic series of slits to project a spatially varying light pattern onto the surface of the skin of healthy volunteers (N=198) at multiple anatomical locations. The backscattered signal was detected with two different setups that used CCD cameras, diffraction gratings, and lenses that were optimized for different wavelength regimes (450 to 800 nm; 950 to 1600 nm). Average values of the absorption and scattering coefficients [($\mu_{a}(\lambda )$, $\mu_{s}(\lambda )$] over the imaged regions (Fig. 3) were compared among anatomical locations, ages, and genders of study participants. The measured spectra showed characteristic absorption peaks of water and lipids not observable in the VIS-NIR, as well as an apparent continuation of the characteristic inverse-power-law form of the scattering spectrum from the VIS-NIR into the SWIR. It is important to note that explicit measurement of $\mu_{s}(\lambda )$, as reported in Ref. 17, is not typically achieved for biological tissues because it requires knowledge of the anisotropy parameter g(λ). The authors of Ref. 17 estimated $\mu_{s}(\lambda )$ by assuming a spectrally constant value of g to be known for their study, based on previous measurements and modeling by their group.^25^

## 3 *In Vivo* SWIR Spectroscopy and Imaging: Diagnosing and Monitoring Burns

Currently, the standard of care to assess burn depth is to rely on subjective assessment by the burn surgeon. However, this assessment is only correct around 60% of the time.^26^^,^^27^ Thus, around 40% of burn patients are either (i) incorrectly diagnosed with a deep burn, receiving unnecessary surgery, or (ii) are incorrectly diagnosed with a shallow burn and do not receive the necessary surgery, which can lead to infection, sepsis, wound healing complications, and debilitating scar contractures.^28^^,^^29^ Viable cells in the human body consist of 70% water, which decreases by 30% during cell death.^30^ Thus, the ability to quantify tissue hydration *in vivo* has the potential to inform tissue viability post-burn. In addition, the sweat and apocrine glands that provide moisture to the skin surface are at the level of the hair follicle bulge, where stem cells necessary for skin regeneration reside (deep dermis). Thus, burns deep to these glands and the skin stem cells result in dehydrated tissue that is unable to regenerate.

A small portion of the SWIR spectral region was included along with the NIR (650 to 1080 nm) in a 2006 study by Sowa et al.^31^ to distinguish superficial and intermediate partial-thickness burns from deep partial-thickness and full-thickness burns in 5 pigs. Fiber-optic spectroscopy was employed to detect the backscattered reflectance of these thermal contact burns, and information from the first derivative of the measured reflectance spectra was used in a classification model that employed partial least squares logistic regression to obtain a percent likelihood of the burn being deep. A cross-validation method that trained the classification algorithm on the data from four of the five animals and used the remaining animal as the test set, iterated over all five animals, was used to determine the accuracy of the model. When the condition for a deep burn diagnosis was defined by a deep burn probability value of greater than 50%, the sensitivity and specificity of the algorithm were 0.9 and 0.83, respectively, for distinguishing deep burns from superficial burns. Using a range of different threshold values on the deep burn probability for a deep burn diagnosis, the area under the resulting receiver operating characteristic (ROC) curve was 0.879. Interestingly, although the 650 to 1080 nm range used in this study demonstrated substantial promise for providing spectral features of burns that could distinguish among different burn depths, the study states that reflectance data were actually obtained over a much wider wavelength range (650 to 2500 nm), but the majority of the SWIR data was not used in the analysis, perhaps indicating that, at this time, it was more difficult to obtain high-quality data over the entire SWIR range.

More recent studies^32^^–^^35^ have utilized a much wider range of SWIR light to provide quantitative burn depth diagnostics. Yin et al.^33^ used SWIR reflectance data from *ex vivo* porcine skin tissue (from five miniature Bama pigs) to train a support vector regression (SVR) algorithm to calculate the numerical values of the depths of thermal contact burns. A broadband lamp and a fiber-optic spectrometer provided data from 900 to 2200 nm. Measured reflectance spectra were fit with a mathematical model to extract parameters related to tissue hemoglobin fraction, water fraction, scatterer size, and scatterer concentration. These parameters were then used as inputs to the SVR algorithm. The algorithm was trained and tested using 10 different combinations of samples, split into training and testing sets in a roughly 2:1 ratio (1037 samples in each iteration of the training set; 520 samples in each iteration of the test set). The predicted burn depths were compared with histology data (Masson trichrome stain). The burn depths predicted by the SVR algorithm were in excellent agreement with those measured via histology, with a percentage error of <14% for all but the shortest burn contact time, and a percentage error of <2% for the three longest burn contact times. These results suggest that the numerical depth of burns can be determined with high accuracy via SWIR reflectance spectroscopy; however, as this study was performed on *ex vivo* tissue, it is unclear whether the technique would be as accurate in dynamic living tissue *in vivo*.

**Fig. 4:**
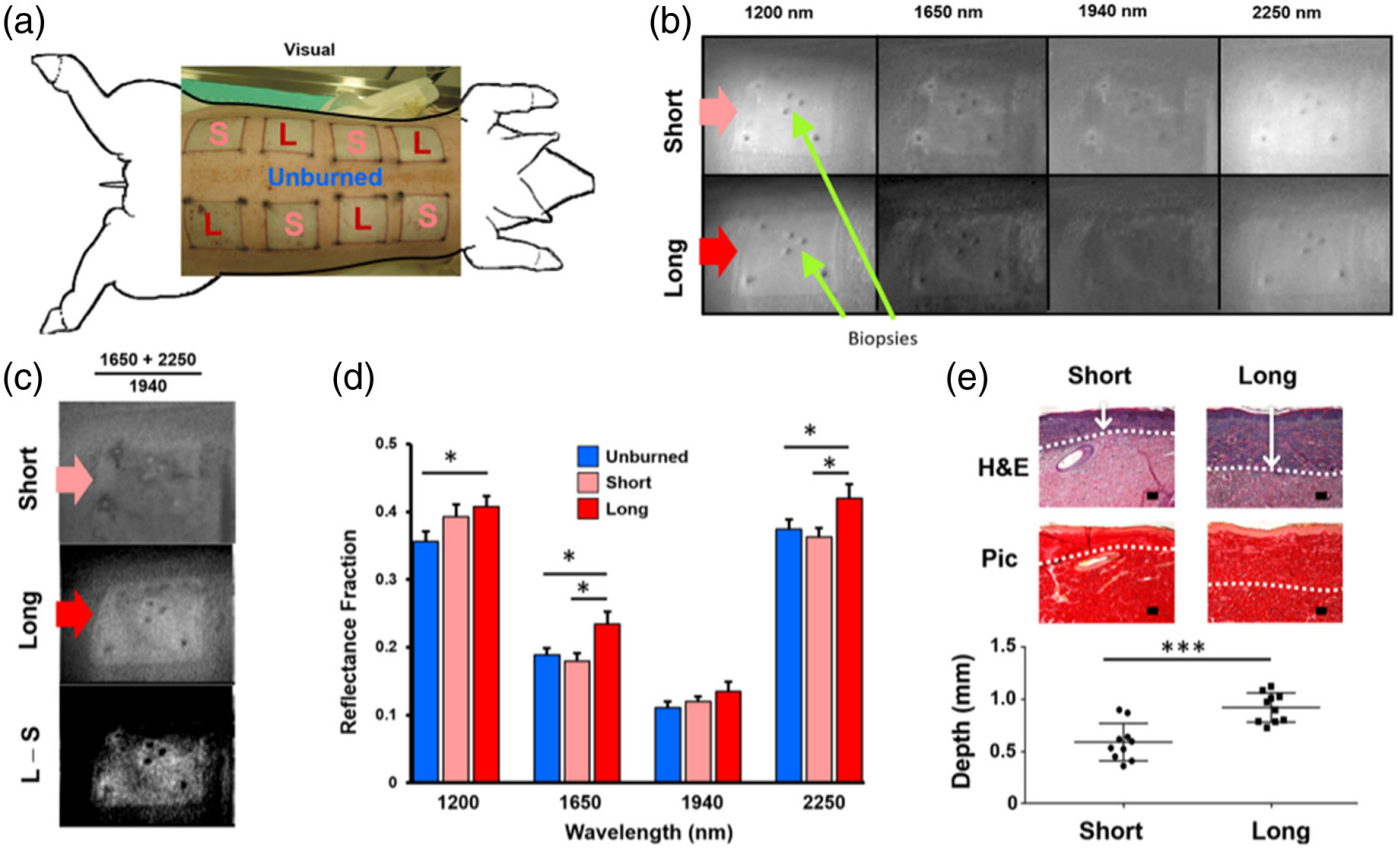
Multispectral SWIR imaging can distinguish between superficial and deeper burns *in vivo*. (a) Thermal contact burns of short (S) and long (L) contact times were produced *in vivo* in a porcine model. (b) SWIR reflectance images at four wavelength bands (1200, 1650, 1940, and 2250 nm) provided contrast between burns and surrounding tissue. (c) A ratio composed of the sum of the 1650 and 2250 nm reflectance images divided by the 1940 nm reflectance image was found to enhance contrast between the short-contact-time and long-contact-time burns. (d) The measured reflectance at 1650 and 2250 nm was significantly different between the short and long contact time burns. (e) Histology confirmed a significant difference between the burn depths produced by the short versus long contact times. Figure reproduced from Ref. 34 with permission; © 2019 by the Wound Healing Society.

Mironov et al.^34^ used broadband light with four SWIR filters (1200, 1650, 1940, 2250 nm) and a SWIR camera to image reflectance from thermal contact burns *in vivo* in a porcine model, demonstrating a correlation between the burn depths obtained from the reflectance data and the burn depths measured via histology (Fig. 4). Two different burn depths were created by changing the contact time of the tool used to generate the thermal burns. Images were acquired 72 h post-burn. A reflectance fraction, defined as R(1650  nm)+R(2250  nm)/R(1940  nm), was used for enhancing the contrast due to moisture to better visualize differences between the superficial and deeper burns. The reflectance averaged over regions roughly 5  mm×5  mm in area for multiple burn sites was used to obtain values characteristic of unburned skin, superficial burns, and deep burns. These averaged reflectance values were found to be significantly higher for the deeper burns (n=17 sites) than the more shallow burns (n=17 sites) at 1650 and 2250 nm.

**Fig. 5:**
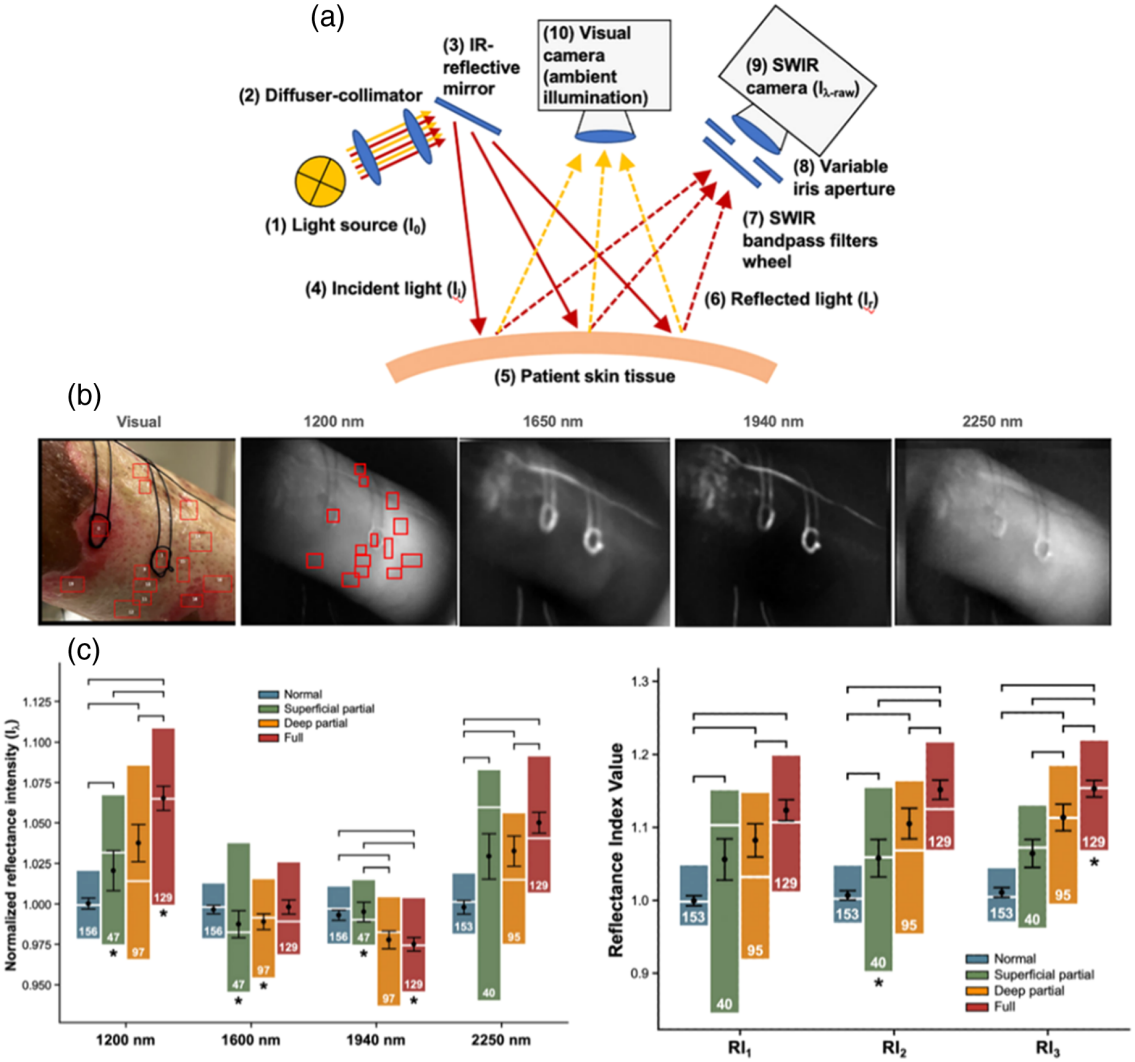
Multispectral SWIR reflectance of human burns can distinguish between different burn depths *in vivo*. (a) Instrumentation for acquiring multispectral SWIR reflectance images, utilizing a broadband light source, four different bandpass filters selecting different wavelength ranges of SWIR light (bands centered at 1200, 1650, 1940, and 2250 nm), and a camera sensitive to SWIR wavelengths. (b) SWIR images of burned tissue *in vivo* in a patient, with regions of interest used for evaluating reflectance data shown in the image taken at 1200 nm (red boxes). (c) Reflectance values at multiple SWIR wavelength bands, normalized to those of surrounding unburned tissue (left), as well as reflectance indices calculated using the SWIR reflectance data (right), demonstrated statistical significance for distinguishing between different severities of burns. Figure panels reproduced and adapted from Ref. 35 under a CC-BY license.

Nunez et al.^35^ applied techniques developed in Ref. 36 to *in vivo* human studies (N=11 patients). As in Ref. 34, four wavelength bands (1200, 1650, 1940, and 2250 nm) were selected via filters for SWIR reflectance imaging to obtain information about burn depth. Reflectance data for multiple regions of interest on the burns were divided by data from unburned tissue, and the resulting data were used to construct three quantities termed reflectance indices (Fig. 5). Burn depth (superficial partial thickness, deep partial thickness, or full thickness, as identified by multiple pathologists and surgeons) was positively correlated with each of the reflectance indices measured via SWIR imaging and accurately identified which burns were deep enough to require surgical intervention. By contrast, the burn depth classifications provided by the pathologists for biopsied tissue sites typically did not agree with those of the surgeons (disagreeing over 60% of the time), indicating how SWIR reflectance has strong potential to fill an unmet need for more accurate and reliable burn severity diagnosis. One reason SWIR is hypothesized to allow for enhanced diagnostic accuracy over other technologies is its sensitivity to tissue moisture.

## 4 *In Vivo* SWIR Spectroscopy and Imaging: Quantifying Tissue Lipid Content

As lipids have absorption peaks around 1200, 1400, and 1750 nm, and each of those peaks is substantially higher than the NIR lipid absorption peak near 930 nm, it is expected that using SWIR wavelengths should provide greater sensitivity to lipid content. This concept has recently been illustrated using both tissue-simulating phantoms and *in vivo* data.^36^^,^^37^

**Fig. 6:**
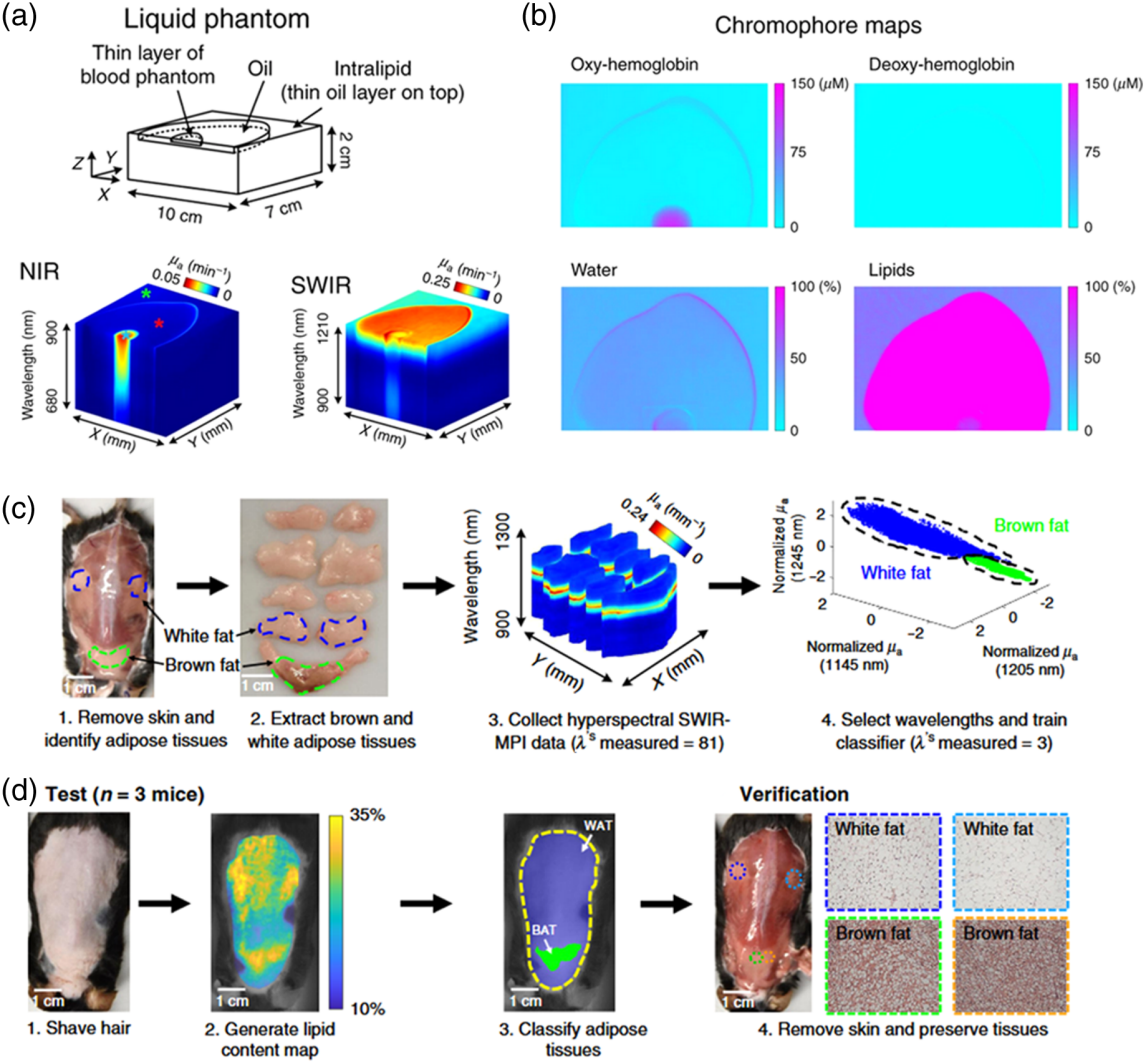
Hyperspectral SWIR imaging quantifies lipid content in tissue-simulating phantoms and *in vivo*. (a) NIR and SWIR absorption coefficient ($\mu_{a}$) datacubes obtained from a tissue phantom containing Intralipid, with a thin layer of oil that aggregated into an oval-shaped pattern on the surface, as well as a small amount of blood localized to one location on the surface. The main contribution to the NIR $\mu_{a}$ datacube is the absorption spectrum of the hemoglobin in the blood, whereas the SWIR $\mu_{a}$ datacube is highly sensitive to the absorption from lipids in the oil and Intralipid, but notably, far less sensitive to the absorption from the region of blood. (b) The sensitivity of the NIR wavelengths to blood is seen in the oxyhemoglobin map, and the sensitivity of the SWIR wavelengths to water and fat is seen in the water and lipid fraction maps. (c) SWIR data cubes were obtained for *ex vivo* white and brown fatty tissue excised from mice, and these data were employed to train an algorithm for distinguishing between these two types of fat. (d) This algorithm was then applied *in vivo* to three mice, demonstrating the ability to distinguish regions of white fat from regions of brown fat. Figure panels reproduced and modified from Ref. 36 under a CC-BY license.

Zhao et al.^36^ (Fig. 6) showed that for a tissue-simulating phantom consisting of intralipid with a thin layer of oil on top and a small amount of blood in one region on top of the phantom, a hyperspectral imager spatially mapped the absorption coefficient from 680 to 1210 nm and used this information to obtain maps of hemoglobin concentration, water fraction, and lipid fraction. The results demonstrated clear identification of the regions that were primarily comprised of blood versus water versus lipids. Zhao et al.^36^ also applied this technology to distinguish between brown and white adipose tissue in living mice (Fig. 6). The algorithm for classifying these two types of tissue was trained by excising samples of these tissues from seven mice, obtaining hyperspectral images of these samples from 900 to 1300, identifying a combination of three wavelengths (1145, 1205, and 1245 nm) that was ideal for distinguishing the absorption coefficients of these two tissue types, and then training the algorithm on data from those three wavelengths. The algorithm was then tested on three mice *in vivo* by imaging a map of lipid absorption, using that data as inputs to the previously trained algorithm to determine which regions of the image represented brown versus white fat, and comparing the classification results against histology. The results demonstrated that SWIR absorption data at the three aforementioned wavelengths were able to successfully distinguish between brown and white fat *in vivo*. In addition, it is important to note that in this same report, experiments were also performed to show a correlation between the lipid fraction measured with SWIR imaging of excised tumors (grown in mice) and the lipid fraction measured via histological staining of those tumors.

## 5 Emerging SWIR-Based Technologies

As SWIR technology has become more widely investigated in the context of tissue spectroscopy and imaging, a larger range of technologies and applications has been explored. Specifically, there has recently been an increase in the use of SWIR light for hyperspectral imaging,^18^^,^^38^^–^^41^ optical coherence tomography,^42^^–^^45^ photoacoustic imaging,^46^ and photothermal imaging.^47^ In addition, devices have been designed to enable *in vivo* SWIR measurements in harder-to-access parts of the body,^48^ and extension of use cases for SWIR biophotonics devices to a wider range of tissue types, including the eye,^49^^,^^50^ brain,^51^ lymphatic tissue,^52^ and teeth.^42^^–^^44^ As the reduced scattering coefficient $\mu_{s}^{\prime }(\lambda )$ in tissue is known to decrease monotonically with wavelength, and absorption from melanin and hemoglobin is far lower in the SWIR than in the VIS-NIR, certain subsets of SWIR wavelengths may have the potential to provide increased depth of penetration for tissue spectroscopy and imaging devices.^49^^,^^50^^,^^53^ Due in part to interest in leveraging this potential for enhanced penetration depth, a substantial number of recent studies have used SWIR light for fluorescence measurements.^54^^–^^68^ A subset of these studies will be discussed in greater detail below.

### 5.1 Next-Generation SWIR Device Development and Expanded Biomedical Application Space

In a 2022 study by Sheen et al.,^38^ a hyperspectral SWIR imager utilizing a push-broom configuration identified notable alterations to the reflectance spectrum in two different SWIR windows (950 to 1100 nm; 1400 to 1650 nm) in the feet of diabetic peripheral neuropathy (DPN) patients, attributed to alterations in collagen and water caused by DPN. In a subsequent 2025 report, Sheen et al.^39^ improved upon their previous instrumentation design by constructing a multispectral SWIR imager with a ∼40% reduction in form factor and a change to the light sources, to create a more clinic-friendly tool for diagnosing DPN. A FOSIL-WSM algorithm selected seven wavelengths for LEDs that were deemed optimal for improving the detection of DPN: 1100, 1150, 1200, 1300, 1450, 1550, and 1650 nm. Using a custom-made kernel least-squares orthogonal subspace projection algorithm to analyze the data (N=188 diabetic patients) to detect DPN yielded an accuracy of 82.4% and an area of 0.885 under the ROC curve.

Gruensfelder et al.^53^ developed a wearable device for pulsatile blood flow monitoring, using SWIR light (1300 nm) in addition to NIR light (900 nm) to provide sensitivity to water and hemoglobin within blood as a means to quantify hemodilution for identifying postpartum hemorrhage in its early stages. The device was tested in tissue-simulating phantoms mimicking pulsatile blood flow (containing blood from sheep), and signal-to-noise ratio (SNR) assessment was performed in humans. Both wavelengths provided quality SNR and showed potential for identifying hemodilution, indicating that the avoidance of VIS-NIR wavelengths that are more highly absorbed by melanin may enhance pulsatile blood flow-based sensing of hemodilution in patients.

**Fig. 7:**
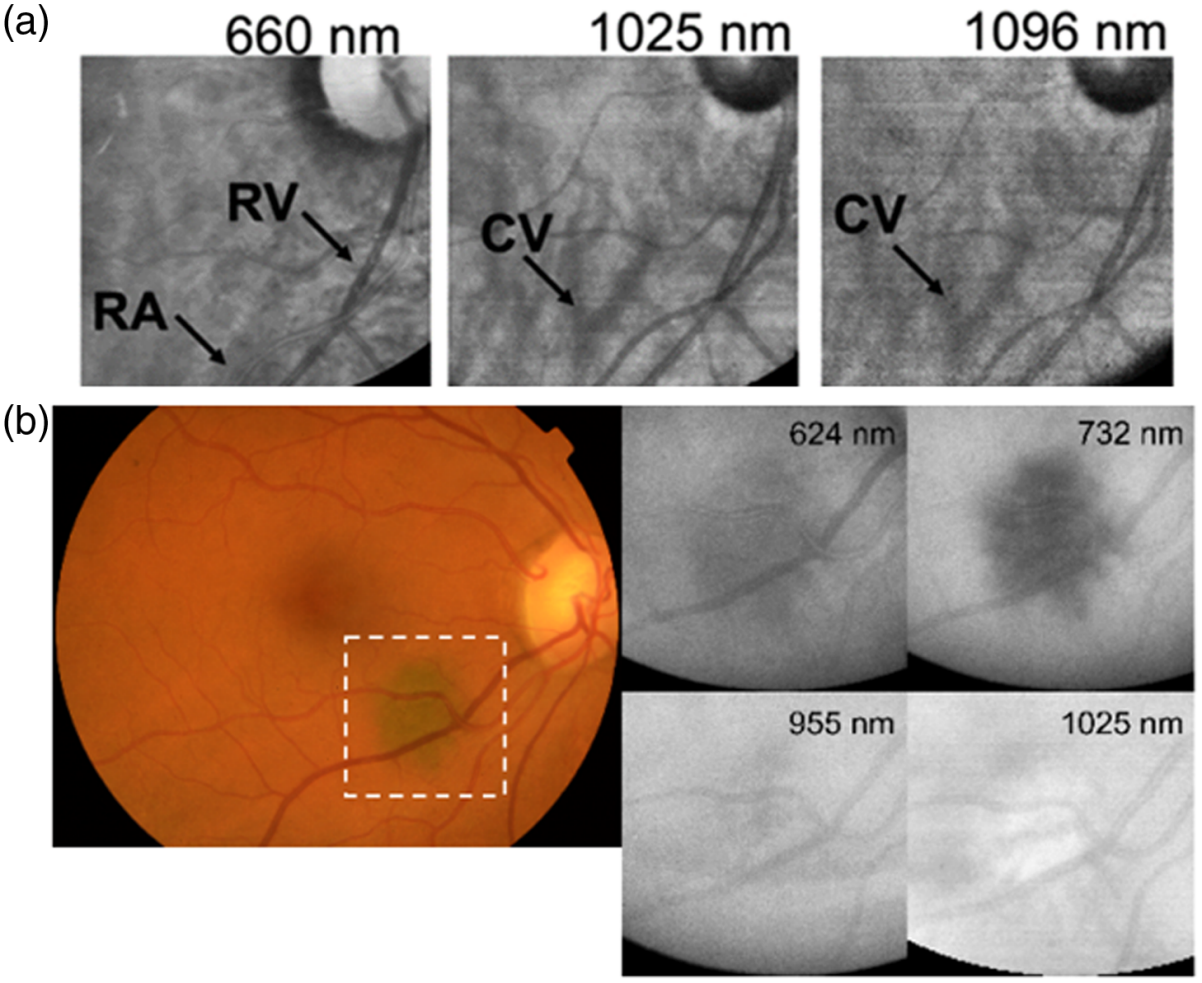
(a) SWIR wavelengths can visualize vasculature deeper beneath the surface of the human eye *in vivo*, as compared to NIR wavelengths, in a custom-built fundus camera. At 660 nm, veins and arteries in the retina (RV; RA) are visible, whereas vessels from the choroid (CV) only become visible when images at SWIR wavelengths (1025 and 1096 nm) are examined. Figure panels from Ref. 49, reprinted and adapted with permission under a CC-BY license. (b) Deeper vasculature in the human eye *in vivo* can be obscured by melanin in the NIR but becomes visible in the SWIR. A choroidal nevus [white dashed box in (A)] appears as a dark spot in multispectral fundus camera images (B) of the human eye at 624 and 732 nm due to the presence of melanin, but the images at 955 and 1025 nm in the region of the nevus are not impacted by absorption from melanin, providing a clearer view of vasculature in regions overlapping with, and beneath, the nevus. Figure panels were reprinted and adapted from Ref. 50 with permission; © Optica Publishing Group.

Alterini et al.^49^ and Burgos-Fernández et al.^50^ reported the development of a fundus camera using SWIR light to penetrate deeper into the retina [Fig. 7(a)]. The camera acquired images of the eye at 15 different wavelength bands within the 400 to 1300 nm range, with a goal of using the increased penetration depth of the SWIR wavelengths to image features of the choroid that would be difficult to observe with VIS-NIR light due to their location within the eye. Specifically, veins and arteries in the retina (RV; RA) are observed at 660 nm, whereas vessels from the choroid (CV) become visible when SWIR wavelengths (1025 nm; 1096 nm) are used for imaging [Fig. 7(a)].

The SWIR fundus camera developed by Alterini et al.^49^ was then employed by Burgos-Fernández et al.^50^ to obtain diagnostic information from different layers of ocular tissue in 167 patients (137 healthy; 30 with conditions affecting the eyes). Reflectance images were obtained at each wavelength and subsequently used for calculating local contrast (LC) and global contrast (GC) ratios. These ratios were employed to distinguish a variety of pathologies (glaucoma, age-related macular degeneration, choroidal nevus) from surrounding tissue, using differences in contrast as a function of wavelength, as well as the combination of different light penetration depths enabled using a device that ranged from VIS-to-SWIR wavelengths. Interestingly, it was the NIR wavelengths that appeared to provide the greatest contrast between diseased and healthy tissue; however, the SWIR wavelengths were able to help identify deeper tissue structures and reduce the obscuring effect of melanin (e.g., from choroidal nevi) on visualization of surrounding vasculature [Fig. 7(b)].

### 5.2 SWIR Fluorescence Imaging

Recently, SWIR wavelengths have been employed to provide contrast for fluorescence imaging as well,^54^^–^^68^ using quantum dots,^55^^–^^62^ carbon nanotubes,^63^ and other materials^64^^–^^68^ that emit fluorescence in the SWIR. These fluorescence studies also leverage the fact that penetration depth at certain SWIR wavelengths may be greater than that in the VIS-NIR due to substantially less absorption from melanin and hemoglobin, as well as monotonically decreasing scattering.

Waterhouse et al.^64^ applied SWIR fluorescence imaging to tumor xenografts *in vivo* in mice. An exogenous contrast agent, Dinutuximab-IRDye800, was injected into the mice; this contrast agent localized in the neuroblastoma cells that had been introduced subcutaneously into the mice. The SWIR imager used a 785 nm laser to excite fluorescence in the exogenous dye. An InGaAs camera, used in conjunction with a sequence of six long-pass filters (850, 950, 1050, 1150, 1250, and 1350 nm), was used for detecting exogenous fluorescence signals from 950 to 1600 nm. Differences between fluorescence spectral lineshapes from tumor and nontumor regions were quantified with principal component analysis (PCA), and separation between these two tissue types was illustrated using data from the first three principal components. Seven different machine-learning-based classification algorithms were compared, and an algorithm combining PCA with a k-nearest neighbor technique demonstrated the highest classification accuracy (97.5% for distinguishing between tumor regions, nontumor regions, and background regions).

A 2024 report by Keizers et al.^65^ compared contrast-to-noise and tumor-to-background ratios between *ex vivo* tumor samples imaged at NIR versus SWIR wavelengths, using an exogenous contrast agent (cetuximab-IRDye800CW). Interestingly, that study did not find a notable advantage of using the SWIR when compared with the NIR. A 2023 study by Privitera et al.^66^ built a device for fluorescence imaging in the NIR and SWIR ranges and applied this device *in vivo.* A comparison of the contrast ratios between tumor tissue and background using NIR versus SWIR wavelengths found that this contrast ratio was enhanced in the SWIR. The discrepancies between the findings of Refs. 65 and 66 may be due to the fact that one study used *ex vivo* tissue, whereas the other study was *in vivo*. The concentrations of endogenous chromophores such as water and hemoglobin (which can attenuate both the excitation light and the emitted fluorescence) are likely to be substantially different between *ex vivo* and *in vivo* tissues, providing confounding variables that make it difficult to compare the results of *ex vivo* and *in vivo* studies of this type. Another potential explanation for the difference in results of these two studies could be the use of different contrast agents. Privitera et al.^66^ conjugated IRDye800CW and IR12 with dinutuximab-beta, which was also used by Waterhouse et al.,^64^ whereas Keizers et al.^65^ conjugated IRDye800CW with cetuximab.

SWIR fluorescence has also recently been incorporated into light-sheet microscopy to image tissue structures *in vivo*.^69^^–^^71^ Using exogenous fluorescence from materials such as lead sulfide/cadmium sulfide quantum dots, imaging at excitation wavelengths of ∼785 to 1320 nm and emission wavelengths of ∼1000 to 1700 nm was achieved. The combination of SWIR wavelengths, light-sheet microscopy technology, and optical clearing (via glycerol) facilitated imaging up to ∼2  mm beneath the surface in an *ex vivo* mouse brain.^69^ The SWIR light-sheet microscope imaged microcirculation, injury response, and distribution of molecules in 3D *in vivo*.^69^ This technology also enabled *in vivo* imaging of the transport of cancer vaccine through the body via the lymphatic system to eliminate tumors and prevent future tumor development.^71^

## 6 Discussion and Conclusion

This review summarizes growing trends over the past decade in the development and use of SWIR spectroscopy and imaging tools for *in vivo* tissue-optics applications. At the time of our previous review,^9^ the use of SWIR wavelengths in biophotonic technology was in its infancy, and thus, the majority of studies up to that point involved *ex vivo* tissue samples. Recently, as SWIR light sources and detectors have become more widely used in the biophotonics field, a proliferation of custom-made devices and biomedical application areas has resulted. Primary goals of these technologies appear to be (1) increasing sensitivity for characterizing and monitoring water and lipid content *in vivo* and (2) accessing information about tissues that are located deeper beneath the surface than those accessed by VIS-NIR devices. SWIR-based spectroscopic imaging devices have used both endogenous contrast (from water and lipid absorption peaks, as well as tissue scattering) and exogenous contrast (from fluorescent dyes that absorb NIR light and emit SWIR light). In addition to the applications detailed in this report, recent studies have been performed to better characterize the penetration depth of SWIR photons,^72^^,^^73^ incorporate SWIR technology into tools for microscopy^69^^–^^71^^,^^73^^,^^74^ and cytometry,^75^ and merge SWIR techniques with machine learning algorithms to facilitate clinical translation.^76^ It is important to note that as the use of SWIR wavelengths for tissue diagnostics is still relatively recent at the time of this report (circa 2025), some key studies presented here are still in the “proof-of-concept” phase, involving small sample sizes. In these cases, future work will likely need to be performed to quantify the degree to which diagnostically relevant changes in SWIR parameters are maintained for larger sample sizes.

The potential for enhanced penetration depth in the SWIR is primarily attributed to (1) the monotonic decrease in scattering as a function of wavelength, and (2) the substantially reduced absorption from melanin and hemoglobin in the SWIR relative to the VIS-NIR. Importantly, however, the window that appears to be most often leveraged for increasing penetration depth in the SWIR is ∼1100 to 1300 nm; for wavelengths longer than this, the absorption from water becomes a major factor in strongly attenuating the detected signal and reducing the penetration depth. Work has begun, and will be important to continue, toward identifying ideal sets of wavelengths throughout the SWIR to facilitate the optimal balance between wide-ranging penetration depths and high sensitivity to small changes in water and lipid content.

There is a tradeoff throughout the SWIR between sensitivity of specific wavelength bands to specific absorbers (e.g., water-related vibrational bands) and “over-sensitivity” to absorption (i.e., absorption being so high at a given wavelength that the detected signal is too low to obtain meaningful information). Wavelengths from ∼1000 to 1350 nm and ∼1600 to 1800 nm are likely optimal for providing sensitivity to absorption from water and lipids while avoiding the prominent absorption peaks of water around 1450 and 1950 nm that often profoundly attenuate the detected signal. The choice of wavelengths can also be influenced by the instrumentation used for performing the measurements. Table 1 provides a comparison between characteristics (materials; wavelength ranges) of light sources and detectors employed for SWIR tissue sensing in selected studies highlighted in this review. Although SWIR tissue sensing technology has evolved substantially during the time period leading up to this review (2016 to 2025), at the time of this review, there are still notable variations between the wavelength ranges of the light sources and detectors employed for SWIR tissue sensing.

**Table 1:** Characteristics of light sources and detectors used for SWIR tissue sensing in selected studies highlighted in this review.

Study	Light source	Detector
Du et al.^13^	Broadband (halogen lamps)	InGaAs cooled CCD camera (Ninox, Raptor) (600 to 1600 nm)
Pilvar et al.^14^	LEDs (880, 1100 nm)	Germanium CCD camera (TriWave; IRLabs) (300 to 1600 nm)
Spink et al.^15^	LEDs (980, 1200, 1300 nm)	InGaAs PIN photodiode (900 to 1700 nm)
Mironov et al.^34^	Broadband (tungsten)	Type 2 strained sensor layer (T2SL) camera (Xevar 2.35-320; Xenics) (1000 to 2350 nm)
Nunez et al.^35^		
Zhao et al.^36^	Tunable pulsed laser (acting as CW source)	Germanium CMOS camera (VIS-NIR-SWIR; exact range not specified)
Carr et al.^67^	Laser (808 nm)	InGaAs camera (NIRvana 640; Princeton Instruments) (900 to 1700 nm)
Wang et al.^69^	Switchable lasers (658, 785, 1319 nm)	InGaAs camera (Princeton Instruments) (900 to 1700 nm)
Ren et al.^71^		

As SWIR techniques for tissue imaging and sensing continue to evolve, another important factor to characterize will be the impact of wavelength on spatial resolution (for imaging) or spatial sensitivity (for point-based methods). As tissue scattering monotonically decreases with wavelength throughout the NIR and SWIR, it may be possible to spatially locate subsurface sources of contrast with more precision in the SWIR than in the NIR. For instance, Carr et al.^67^ recently demonstrated that using a SWIR camera with a long-pass filter to detect indocyanine green (ICG) fluorescence at wavelengths greater than 1300 nm, cerebral vasculature could be visualized *in vivo* in a mouse model with roughly twice the spatial resolution of that obtained using NIR light. However, as the wavelength-dependent decrease in tissue scattering follows an inverse power law that appears to flatten in the limit of the longest SWIR wavelengths, the amount of scattering in the SWIR is likely only ∼15% less than that at the longest NIR wavelengths, as seen in the SWIR scattering spectra of *in vivo* human skin reported by Kono and Yamada.^17^ Therefore, tissue scattering will still place fundamental limits on the spatial resolution of diffuse-light-based SWIR imaging of subsurface tissue structures. As a result, the greatest opportunity for subsurface tissue sensing with micro-scale spatial resolution (at least for the first 1 to 2 mm beneath the surface) using SWIR wavelengths will likely be provided by scattering-rejection techniques such as light-sheet microscopy.^69^ Also of substantial importance will be expanding upon existing work to quantitatively compare the sensitivity of SWIR versus VIS-NIR wavelengths for identifying and monitoring signatures of tissue disease and injury.

## Data Availability

As this is a review paper, there is no code or new data associated with this article.
